# M2 macrophage-derived extracellular vesicles facilitate CD8+T cell exhaustion in hepatocellular carcinoma via the miR-21-5p/YOD1/YAP/β-catenin pathway

**DOI:** 10.1038/s41420-021-00556-3

**Published:** 2021-07-16

**Authors:** Jian Pu, Zuoming Xu, Jiahui Nian, Quan Fang, Meng Yang, Youguan Huang, Wenchuan Li, Bin Ge, Jianchu Wang, Huamei Wei

**Affiliations:** 1 Department of Pathology, Affiliated Hospital of Youjiang Medical University for Nationalities, Guangxi Zhuang, China; 2 Clinic Medicine Research Center of Hepatobiliary Diseases, Guangxi Zhuang, China; 3 Department of Hepatobiliary Surgery, Affiliated Hospital of Youjiang Medical University for Nationalities, Guangxi Zhuang, China; 4 Graduate College of Youjiang Medical University for Nationalities, Guangxi Zhuang, China

**Keywords:** Cancer stem cells, Cancer, Cell biology, Cancer

## Abstract

Hepatocellular carcinoma (HCC) is a common malignancy. CD8^+^ T cell-mediated immune response is critical for the inhibition of HCC progression. M2 macrophages participate in HCC progression. This study set out to investigate the effect of M2 macrophage-derived extracellular vesicles (EVs) on CD8^+^ T cell exhaustion in HCC. M2 macrophage-derived EVs were isolated and identified. The murine model of primary HCC was established through DEN/CCl_4_ induction, and model mice were injected with EVs. Peripheral blood mononuclear cells (PBMCs) were isolated from the mouse liver and CD8^+^ T cells were sorted. The expressions of immune checkpoint inhibitory receptors and effector cytokines on CD8^+^ T cells were detected, followed by the evaluation of CD8^+^ T cell proliferation and killing function. miR-21-5p expression in M2 macrophage-derived EVs was detected. The binding relationship between miR-21-5p and YOD1 was verified. The activation of the YAP/β-catenin pathway was detected. Consequently, M2 macrophage-derived EVs promoted CD8^+^ T cell exhaustion in HCC mice. miR-21-5p expression was upregulated in M2 macrophage-derived EVs, and EVs carried miR-21-5p into HCC tissues. miR-21-5p targeted YOD1. Inhibition of miR-21-5p or overexpression of YOD1 annulled the promoting effect of EVs on CD8^+^ T cell exhaustion. YOD1 inactivated the YAP/β-catenin pathway. In conclusion, M2 macrophage-derived EVs facilitated CD8^+^ T cell exhaustion via the miR-21-5p/YOD1/YAP/β-catenin axis. This study may confer novel insights into the immunotherapy of HCC.

## Introduction

Primary hepatocellular carcinoma (HCC) is an aggressive malignancy that constitutes the primary cause of cancer-related death [1]. The acknowledged risk factors for HCC include chronic HBV or HCV infection, metabolic liver disorders, and liver cirrhosis [2]. The microenvironment of highly immune HCC makes immunotherapy a promising target for HCC [3]. Tumor-induced immunosuppression and immune evasion facilitate cancer progression and impede the efficacy of immunotherapy [4, 5]. Indeed, a large number of immunotherapies have been designed for HCC patients to enhance tumor-specific immune response, but the clinical benefits are limited [6]. Despite certain genetic and epigenetic alterations that have been observed in HCC cells, the exact pathogenesis of HCC is not elucidated [7]. Surgical resection and liver transplant are available for the HCC patients at the early stage; however, the overall survival rate is still limited due to the high recurrence rate and low qualified rate of surgery and transplant [8]. A systematic review of tumor-infiltrating lymphocytes demonstrates that the major reasons for tumor escape in the immune system include the dysfunction of CD8^+^ T cells and the presence of excessive suppressor T cells [9]. Under the condition of prolonged antigen exposure, the tumor-specific effector CD8^+^ T may differentiate into a stage called T cell exhaustion [10]. CD8^+^ T cell exhaustion is often concerned with invalid infection control and the weakened anti-tumor effect [11]. Hence, targeting CD8^+^ T cells is the main direction of immunotherapy for HCC.

Tumor microenvironment (TME) is composed of various cell types such as endothelial cells, fibroblasts, and immune cells, and extracellular components such as growth factors, proteolytic enzymes, extracellular matrix, and cytokines [12]. All cellular and non-cellular components of TME generate a neoplastic niche where tumor cells proliferate rapidly and escape the attack of the host defense system [13]. Macrophages are the most prominent immune cells in TME. Macrophages are a class of leukocytes with antigen presentation ability, which actively participate in tissue remodeling, phagocytosis, and clearance of foreign substances and cell debris [14]. Macrophages have a variety of functions related to cancer progression; they contribute to the escape of tumor cells and hinder the anti-tumor immune mechanism and response [15]. Macrophages are categorized into the classically activated (M1) and the alternatively activated (M2) macrophage according to the polarization status; M1 macrophages exert tumoricidal effects while M2 macrophages facilitate tumorigenesis [16]. In TME, M2 macrophages have tumor-promoting activities by facilitating HCC cell proliferation, migration, angiogenesis, and immunosuppression, and additionally, M2 macrophages are associated with the unfavorable outcome of HCC [17]. Extracellular vesicles (EVs) are bioactive molecular shuttles packaged by proteins, lipids, and nucleic acids, which modulate TME by interacting with adjacent cells [18]. M2 macrophage-derived EVs can lead to the progression of colon cancer [19] and gastric cancer [20]. However, the effect of M2 macrophage-derived EVs on CD8^+^ T cell exhaustion in HCC remains unknown. This study herein investigated the effect of M2 macrophage-derived EVs on CD8^+^ T cell exhaustion in HCC, which shall confer novel insights into the immunotherapy of HCC.

## Materials and methods

### Ethics statement

This study was approved by the Ethical Committee of Affiliated Hospital of Youjiang Medical College for Nationalities. Adequate laboratory procedures were made to minimize the pain of mice, such as heating pads, disinfection, and fluid replacement with normal saline.

### Isolation and identification of mouse macrophage

Female C57BL/6 mice (aged 6–8 weeks and weighing 20–24 g) were purchased from DOSSY Experimental Animals Co., Ltd (Chengdu, Sichuan, China) [SCXK (Sichuan) 2019-028] and euthanized by intraperitoneal injection of ≥100 mg/kg pentobarbital sodium. Two femurs of each mouse were separated and soaked in 75% alcohol for 5 min. The femurs were washed twice with sterile phosphate-buffered saline (PBS). The two ends of the femurs were cut off, and then the bone marrow in the femurs was repeatedly rinsed with Dulbecco’s modified Eagle’s medium (DMEM) by a syringe until the femurs turned white. The rinsed bone marrow cell suspension was collected and centrifuged at 150 g for 5 min to remove the supernatant. The cells were resuspended in DMEM. The isolated bone marrow cells were seeded on a 10-cm cell culture plate and cultured in DMEM/F12 complete medium containing 10% fetal bovine serum (FBS). Macrophage colony-stimulating factor (M-CSF) was added to the medium till the concentration reached 100 U/mL. The medium was refreshed every 3 days and supplemented with M-CSF. The non-adherent cells were removed and bone marrow-derived macrophages were obtained after 7 days.

Bone marrow-derived macrophages were harvested, suspended in 5 μL PBS and dripped on the slides. After drying, the macrophages were stained with Wright’s dye for 10 min, washed with running water, and observed under the microscope. Then, 1 × 10^6^ cells were collected and resuspended for flow cytometry. The macrophages were cultured with two fluorescent antibodies and homologous control on ice for 30 min, washed with fluorescence-activated cell sorting buffer, and fixed in 10% formalin. The positive rate of antigen was detected using flow cytometer. The antibodies used were CD68 (1:100, ab31630, Abcam, Cambridge, MA, USA) and CD163 (1:60, ab182422, Abcam).

### Induction and identification of M2 macrophage

The mouse bone marrow-derived macrophages were seeded into the 6/12/24 well plates and cultured in DMEM/F12 complete medium containing 10% FBS. After cell adherence to the wall, the cells were treated with 20 ng/mL IL-4 (Sigma-Aldrich, Merck KGaA, Darmstadt, Germany) for 24 h [21]. The expressions of typical polarized molecules CCL22 and PPARγ in M2 macrophages were detected using RT-qPCR. The positive rates of CD163 and CD86 antigens were detected using flow cytometry.

### Isolation and identification of M2 macrophage-derived EVs

EVs in FBS were removed after centrifugation at 100,000 × *g* for 10 min. When M2 macrophages reached about 80% confluence, the supernatant was removed. Then M2 macrophages were added with 10% EVs-free FBS and cultured at 37 °C for 48 h. M2 macrophage-derived EVs were extracted by ultracentrifugation [22]: the supernatant was collected when the medium was refreshed, and then centrifuged at 4 °C and 500 g for 10 min and at 12000 × *g* for 20 min. The supernatant was filtered through a 0.22 μm filter membrane and centrifuged at 100,000 × *g* for 2 h. The precipitates were resuspended in PBS, ultra-centrifuged again for 2 h, and stored at -80 °C after PBS resuspension. The EVs derived from the isolated M2 macrophages were identified by the following methods: the morphology of isolated EVs was observed under the transmission electron microscope (TEM); the size distribution of the EVs was analyzed using Nanoparticle tracking analysis (NTA); the expressions of CD9, CD63, and CD81 on the surface of EVs were verified using Western blotting, with the supernatant of M2 macrophages added with GW4869 as negative control (NC). The identified EVs were lysed, and the total protein content was determined using a bicinchoninic acid (BCA) assay kit. The protein content was used as the standard for EVs.

EVs were assigned into 4 groups: NC group (supernatant of M2 macrophages added with GW4869), EVs group, EVs-NC group (EVs were isolated from inhibitor NC-transfected M2 macrophages), and EVs-inhi group (EVs were isolated from miR-21-5p inhibitor-transfected M2 macrophages). miR-21-5p inhibitor and inhibitor NC were provided by GenePharma (Shanghai, China).

### Establishment of the murine model of primary HCC

Two weeks old C57BL/6 mice were intraperitoneally injected with diethylnitrosamine (DEN; 25 mg/kg) once a week for 3 consecutive weeks. When the mice were 5 weeks old, CCl_4_ (0.5 μL/g) was intraperitoneally injected once a week for 12 consecutive weeks. At the 15^th^ and 40^th^ weeks after induction, the liver of mice was detected.

The experimental mice were assigned into 6 groups: control group, HCC group, HCC + NC group (mice were injected with NC via tail vein), HCC + EVs group (mice were injected with EVs via tail vein), HCC + EVs-NC group (mice were injected with EVs-NC via tail vein), HCC + EVs-inhi group (mice were injected with EVs-inhi via tail vein). According to the protein content of EVs, the mice were injected with 20 μg EVs every day for 7-20 days. All mice were euthanized by intraperitoneal injection of ≥ 100 mg/kg pentobarbital sodium, followed by tissue collection. In this study, 108 mice were used for the establishment of the murine model of primary HCC, with 18 mice in each group. Among them, 6 mice were used for tumorigenesis observation, 6 mice were used for hematoxylin and eosin (HE) staining to observe the histopathological changes and immunohistochemistry to detect the number of CD8^+^ T cells, and the remaining 6 mice were used for the extraction of peripheral blood mononuclear cells (PBMCs) and determination of miR-21-5p and YOD1 mRNA expression.

#### Hematoxylin-eosin (HE) staining

The liver tissues of mice were fixed with 4% formaldehyde for 6 h and embedded in paraffin. The paraffin-embedded liver tissues were cut into 3 μm sections, baked overnight at 60°C, and dewaxed twice with xylene I (14936-97-1, Shanghai Research Biological Technology Co., Ltd., Shanghai, China) and xylene II (523-67-1, Shanghai Yuduo Biotechnology Co., Ltd., Shanghai, China). The sections were soaked in 100%, 100%, 95%, 80%, and 70% ethanol for 5 min and then put into distilled water. Next, the sections were stained with hematoxylin (474-07-7, Qingdao Jisskang Biotechnology Co., Ltd., Qingdao, China) for 10 min and washed with running water for 15 min to turn sections blue. Then, the sections were stained with eosin (RY0648, Qingdao Jisskang Biotechnology co., Ltd.) for 30 s, washed with double-distilled water, dehydrated with alcohol, cleared with xylene, sealed with neutral balsam, and analyzed using a morphological image analysis system (JD801, Jeda Technology Co. Ltd., Nanjing, China).

#### Immunohistochemistry (IHC)

Immunohistochemical assay was performed using diaminobenzidine (DAB) IHC kit (GS4974, Biolab, Beijing, China) to detect the number of CD8^+^ T cells in liver tissues. The liver tissues of HCC mice were baked at 45 °C for 3 h. After dewaxing and hydration, the sections were added with 3% H_2_O_2_ and placed at room temperature for 10 min to eliminate the activity of endogenous peroxidase. Following 3 times of PBST washing, the sections were put into citrate buffer for microwave antigen repair. Next, the solution was heated to boiling and cooled naturally (5–10 min). After 3 times of repair, it was cooled to room temperature. Then, the sections were incubated with goat serum at 37 °C for 1 h, and then the blocking buffer was removed. Next, the sections were incubated with the primary antibody CD8 (1:2000, ab217344, Abcam) at 4 °C overnight. The next day, the sections were rewarmed to room temperature, washed with PBST 3 times, and incubated with biotinylated secondary antibody working solution at 37 °C for 15 min. The sections were washed 3 times with PBST to wash out the unbound antibody and incubated with HPR-labeled streptavidin working solution at 37 °C for 15 min. Following 3 times of PBST washing, the sections were incubated with DAB chromogenic solution for 5 min at room temperature, counterstained with hematoxylin, and then dehydrated, cleared, and sealed.

### Extraction of mouse liver PBMCs

The mice were euthanized, and the liver was collected and cut up and ground into homogenate by adding PBS, and then transferred to a 50 mL centrifuge tube for 1-min centrifugation at 150 g to remove the supernatant. After centrifugation at 400 g for 5 min, the supernatant was removed and the cell precipitate was resuspended using PBS. After centrifugation at 400 g for 5 min, the supernatant was discarded and the sample was washed with PBS once. The samples were added with 5 mL 40% percoll working solution for resuspension and centrifuged at 800 g for 25 min. The upper two layers were rapidly isolated and the lower precipitate was collected. Then, 3 mL red blood cell lysate was added to resuspend the cells. The cells were lysed at 4°C for 10 min and added with 20 mL PBS to terminate the lysis. The cells were centrifuged at 400 g for 5 min to remove the supernatant and resuspended in an appropriate amount of PBS. Afterwards, the cells were filtered through a 200-mesh screen and finally the liver PBMCs were obtained.

### CD8^+^ T cell sorting and culture

PBMCs prepared from liver tissues were filtered through a cell filter (70 μm), added with the upper layer of Ficoll-Paque (10 mL), and centrifuged at 400 g and 20°C for 30 min. After centrifugation, the cloud-like lymphocyte layer in the middle was further sorted using CD8^+^ magnetic beads. The cells that need to be analyzed using flow cytometer were pretreated. Fixable viability dye (FVD) was used to remove dead cells. The cell staining buffer containing 2% FBS and 2 mM ethylene diamine tetraacetic acid was used for the detection of cell surface markers. The purity (>95%) of sorted cells was determined using flow cytometry. The sorted CD8^+^ T cells were stimulated using anti-CD3 (5 μg/mL) and CD28 (2 μg/mL) antibodies in vitro.

CD8^+^ T cells were assigned into 6 groups: blank group [partially exhausted CD8^+^ T cells (PD1^int^ TIM3^+^) were sorted from mouse HCC infiltrating CD8^+^ T cells], EVs group (CD8^+^ T cells were treated with 50 μg EVs for 6 h), EVs-NC group (CD8^+^ T cells were treated with 50 μg EVs-NC for 6 h), EVs-inhi group (CD8^+^ T cells were treated with 50 μg EVs-inhi for 6 h), oe-YOD1 + EVs group (CD8^+^ T cells were transfected with pcDNA-YOD1 for 24 h and then treated with 50 μg EVs for 6 h) and oe-NC + EVs group (CD8^+^ T cells were transfected with pcDNA-NC for 24 h and then treated with 50 μg EVs for 6 h).

### Flow cytometry

The total number and concentration of sorted CD8^+^ T cells were calculated using a cell counter. For cell surface staining, the cells were resuspended in 2% PBS, cultured with primary antibodies [PD1 (17-9981-82, eBioscience, San Diego, CA, USA), TIM3 (119717, BioLegend, San Diego, CA, USA), IFN-γ (564336, BD Bioscience), IL-2 (562483, BD Bioscience) and TNF-α (25-7321, eBioscience)] on ice for 30 min and washed twice with PBS, followed by detection on flow cytometer. For intracellular staining, the cells were fixed, permeabilized, and cultured with fluorescence-labeled secondary antibody for 30 min, and finally detected on flow cytometer. For the detection of cytokines, CD8^+^ T cells were stimulated by 50 ng/mL phorbol myristate acetate (PMA) (Enzo, NY, USA), 1 μg/mL ionomycin (Enzo), and 10 μg/mL brefeldin A (Enzo) at 37°C for 6 h and then treated with the corresponding dyeing. The dead cells were labeled with Fixable Viability Stain 620 reagent (BD Biosciences) and deleted during flow cytometry. FACS Aria II Cell Sorter (BD Biosciences) was used for flow cytometry and FlowJo software (TreeStar, Ashland, USA) was used for data analysis.

### Detection of CD8^+^ T cell killing

The sorted CD8^+^ T cells were activated using anti-CD3 (5 μg/mL) and CD28 (2 μg/mL) for 3 days. Mouse hepatoma cell line Hepal-6 [Cell Bank of Chinese Academy of Sciences (Shanghai, China)] was labeled with carboxyfluorescein diacetate succinimidyl ester (CFSE) and then seeded on the 48-well plates (2 × 10^5^ cells/well). The activated CD8^+^ T cells were cultured with adherent hepatoma cells overnight at an effector-target ratio of 20:1. The cell mixture was collected and the dead cells were stained with 7AAD (A1310, Invitrogen Inc., Carlsbad, CA, USA) for 10 min. The cell killing was measured using flow cytometry: killing efficiency (%) = (% CFSE^+^ 7AAD^+^)/(% CFSE^+^) × 100.

### Measurement of CD8^+^ T cell proliferation

The sorted CD8^+^ T cells were activated using anti-CD3 (5 μg/mL) and CD28 (2 μg/mL) antibodies for 3 days. CD8^+^ T cell proliferation was measured using flow cytometry.

### Uptake of EVs

Antigen-specific CD8^+^ T cells were seeded onto the 24-well cell slides (3 × 10^4^ cells/well). After cell adherence, the cells were cultured with PKH67 (Sigma-Aldrich)-labeled EVs (80 g/mL) for 1 day. The slides were fixed in 4% paraformaldehyde for 20 min, washed 3 times with PBS, stained with 4′,6-diamidino-2-phenylindole for 1 h, sealed with anti-fluorescence quenching agent, and photographed under the fluorescence microscope.

### Reverse transcription quantitative polymerase chain reaction (RT-qPCR)

The total RNA was extracted using TRIzol reagent (Invitrogen) and reverse transcribed into cDNA using PrimeScript RT reagent kit (Takara, Dalian, China). The TaqMan primers and probes were all from TaKaRa (Kyoto, Japan). The ABI PRISM 7900 sequence detection system of SYBR Green II (TaKaRa) was used for quantitative PCR. The PCR procedures were as follows: pre-denaturation at 95°C for 5 min, and 40 cycles of denaturation at 95 °C for 15 s, annealing at 60 °C for 20 s and extension at 72 °C for 35 s. GAPDH and U6 were used as the internal reference. The relative expression of genes was calculated using the 2-^ΔΔCT^ method. The primer sequences (Sangon Biotech, Shanghai, China) are shown in Supplementary Table 1.

### Western blotting

The liver homogenate and cells were lysed in enhanced radio-immunoprecipitation assay lysate containing protease inhibitor for 20 min and centrifuged at 4 °C for 20 min to collect the supernatant. The protein concentration in the supernatant was detected using the BCA protein quantitative kit (Boster Biological Technology Co., Ltd, Wuhan, Hubei, China). The proteins were separated by 10% SDS-PAGE and transferred onto polyvinylidene fluoride membranes. The membranes were blocked with 5% bovine serum albumin for 2 h to block nonspecific binding. Next, the membranes were incubated with the primary antibodies YAP1 (ab56701, Abcam) and β-catenin (ab32572, Abcam) at 4 °C overnight. Following tris-buffered saline-tween washing, the membranes were incubated with HRP-labeled secondary antibody for 1 h and developed with enhanced chemiluminescence reagent (Merck Millipore, Billerica, MA, USA). The gray level of each band was analyzed using Image Pro Plus 6.0 (Media Cybernetics, Bethesda, MA, USA), with β-actin (ab8226, Abcam) as an internal reference. The experiment was repeated three times.

### Bioinformatics analysis

The target genes of miRNA were predicted through Starbase (http://starbase.sysu.edu.cn/), RNAInter database (http://www.rna-society.org/rnainter/) and miRDB (http://www.mirdb.org/). The coexpression relationship of genes was searched through Coexpedia (http://www.coexpedia.org/), and the coexpression score was obtained to further screen the target genes.

### Statistical analysis

Data analysis was introduced using the SPSS 21.0 (IBM Corp., Armonk, NY, USA) and GraphPad Prism 8.0 (GraphPad Software Inc., San Diego, CA, USA). Kolmogorov-Smirnov test was used to verify the normal distribution of continuous variables. Data are expressed as mean ± standard deviation. The *t*-test was used for comparison between two groups. One-way analysis of variance (ANOVA) was employed for comparisons among multiple groups, followed by Tukey’s multiple comparisons test. The value of *p* < 0.05 indicated a significant difference.

## Results

### Isolation and identification of M2 macrophage and EVs

Macrophages were obtained from the bone marrow of mice and stained with Wright after primary culture. Under the microscope, the morphology of macrophages was irregular, and the nuclei were in dark blue and inclined to one side, showing typical morphological characteristics of macrophages (Supplementary Fig. 1A). Flow cytometry showed that macrophage-specific molecular marker CD68 was positive (Supplementary Fig. 1B). M2 macrophages were prepared by IL-4 induction. M2 macrophages were significantly enlarged under the microscope, mainly presented as large round cells (Supplementary Fig. 1C). RT-qPCR showed that the mRNA expressions of CCL22 and PPARγ in M2 macrophages were notably increased after induction (Supplementary Fig. 1D, E). Flow cytometry indicated that the number of CD163^+^ cells (M2 macrophages) was over 90%, and the number of CD86^+^ cells (M1 macrophages) was little (Supplementary Fig. 1F), indicating that M2 macrophages were successfully induced and obtained. EVs were isolated from M2 macrophages by ultracentrifugation. Under the TEM, EVs were round or oval with uneven particles and were 60–160 nm in diameter, and the membrane-like structure was observed around the EVs (Supplementary Fig. 1G). NTA further confirmed the size and distribution of EVs. EVs were mainly distributed at about 100 nm, and the concentration was 2.3 × 10^6^ particles/mL (Supplementary Fig. 1H). Western blotting demonstrated that CD9 and CD63 were enriched in the extracted EVs, while there was no significant expression of calnexin (Supplementary Fig. 1I). Briefly, the M2 macrophage-derived EVs were successfully isolated.

### M2 macrophage-derived EVs promoted tumor progression and CD8^+^T cell exhaustion in mice with primary HCC

**Fig. 1:**
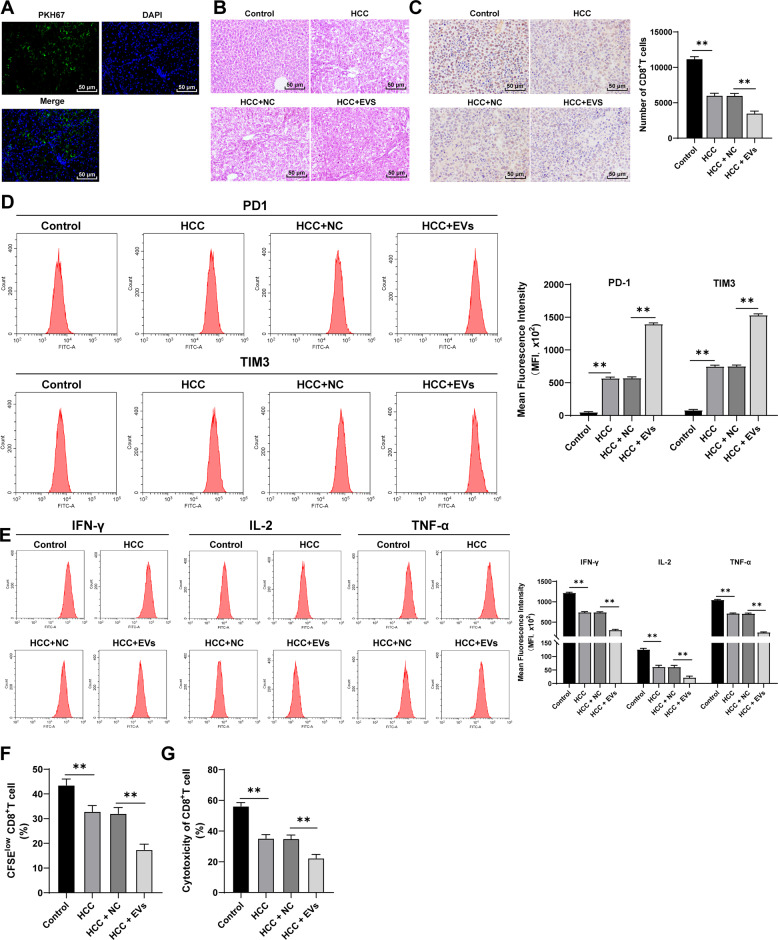
M2 macrophage-derived EVs promoted CD8^+^ T cell exhaustion in mice with primary HCC. The murine model of primary HCC was established by DEN/CCl_4_ combined induction, and the model mice were injected with EVs via tail vein. **A** The absorption of EVs by mouse liver tissues was detected using immunofluorescence. Green indicated PKH67-labeled EVs and blue indicated nuclear staining. **B** Histopathological changes of liver tissues were observed using HE staining at the 40^th^ week after induction. **C** The number of CD8^+^ T cells in tumor sections was detected using immunohistochemistry. PBMCs were isolated from the mouse liver and CD8^+^ T cells were sorted. **D** The expressions of immune checkpoint inhibitory receptors (PD1 and TIM3) on CD8^+^ T cells were detected using flow cytometry. **E** The expressions of effector cytokines (IFN-γ, IL-2, and TNF-α) were detected using flow cytometry. **F** The proliferation ability of CD8^+^ T cells was measured using flow cytometry. **G** The ability of CD8^+^ T cells to kill Hep1-6 cells was measured using flow cytometry. *N* = 6. The cell experiment was repeated 3 times. Data are presented as mean ± standard deviation and analyzed using one-way ANOVA, followed by Tukey’s multiple comparison test, **p < 0.01.

The murine model of primary HCC was established by DEN/CCl_4_ induction to explore the role of M2 macrophage-derived EVs in HCC. EVs were labeled with PKH67, and the green fluorescence could be observed in the liver tissues, indicating that EVs were transferred into the mouse liver via tail vein injection and absorbed by liver tissues (Fig. 1A). After 40 weeks of induction, the mice developed primary HCC, and obvious tumor nodules were observed on the surface of the liver. EV treatment promoted the tumorigenesis in mice, with a higher malignant degree of tumor (Fig. 1B). Immunohistochemistry showed that compared with that in healthy control mice, the number of infiltrating CD8^+^T cells in HCC mice was significantly reduced, and EV treatment further reduced the number of infiltrating CD8^+^ T cells in HCC tissues (Fig. 1C). Then, PBMCs were isolated from the mouse liver and CD8^+^ T cells were sorted. The expressions of immune checkpoint inhibitory receptors (PD1 and TIM3) on CD8^+^ cells and the production of effector cytokines (IFN-γ, IL-2, and TNF-α) were detected using flow cytometry. Compared with healthy control mice, HCC mice showed notably enhanced levels of surface markers of CD8^+^ T cell exhaustion (all *p* < 0.01; Fig. 1D) and reduced effector cytokines (all *p* < 0.01; Fig. 1E), suggesting that the infiltrating CD8^+^ T cells in HCC mice were in a state of functional exhaustion. To further verify the functional exhaustion of CD8^+^ T cells in the liver, we labeled CD8^+^ T cells with CFSE and stimulated them with anti-CD3/CD28 antibodies for 3 days, and then detected their proliferation and killing function. Compared with that in control mice, the proliferation of CD8^+^ T cells in HCC mice was notably decreased (Fig. 1F), and the ability to kill Hep1-6 cells was weakened (Fig. 1G). The addition of EVs further increased the expressions of surface markers of CD8^+^ T cell exhaustion, decreased the production of effector cytokines, and weakened the proliferation of CD8^+^ T cells and the ability to kill Hep1-6 cells. Altogether, M2 macrophage-derived EVs promoted CD8^+^ T cell exhaustion in mice with primary HCC.

### EVs carried miR-21-5p into the liver tissue of mice

**Fig. 2:**
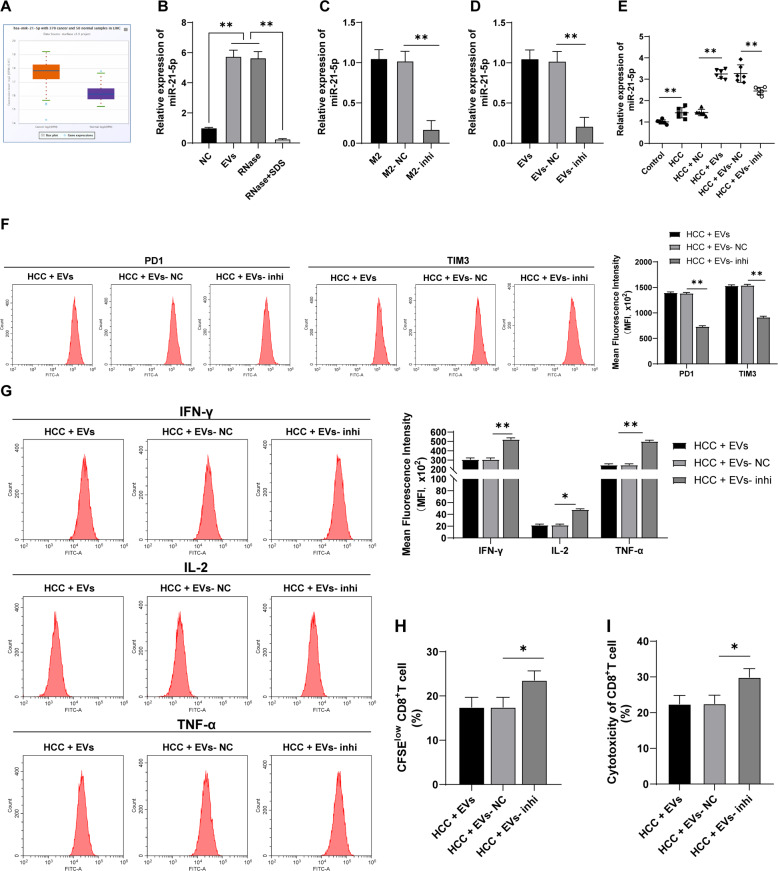
EVs carried miR-21-5p into the liver tissues of mice. **A** miR-21-5p expression in HCC was analyzed through the ECORI Pan-Cer database. **B** miR-21-5p expression in EVs under different treatments was detected using RT-qPCR. M2 macrophages were transfected with miR-21-5p inhibitor. **C**, **D** miR-21-5p expression in M2 macrophages and M2 macrophage-derived EVs was detected using RT-qPCR. **E** HCC mice were injected with EVs under different treatments and the mice were killed after 20 days to obtain liver tissues, and miR-21-5p expression in mouse liver tissues was detected using RT-qPCR. PBMCs were isolated from the mouse liver and CD8^+^ T cells were sorted. **F** The expressions of PD1 and TIM3 on CD8^+^ T cell surface were detected using flow cytometry. **G** The expressions of effector cytokines (IFN-γ, IL-2, and TNF-α) were detected using flow cytometry. **H** The proliferation ability of CD8^+^ T cells was measured using flow cytometry. **I** The ability of CD8^+^ T cells to kill Hep1-6 cells was measured using flow cytometry. *N* = 6. The cell experiment was repeated 3 times. Data are presented as mean ± standard deviation and analyzed using one-way ANOVA, followed by Tukey’s multiple comparison test, *p < 0.05, **p < 0.01.

Lou et al. have analyzed the miRNA microarray GSE69580 of HBV-related HCC and found that miR-21 is abnormally upregulated in HCC and plays a vital role in the prognosis of HCC [23]. ECORI Pan-Cer database also demonstrated that miR-21-5p was highly expressed in HCC (Fig. 2A). Additionally, functional miR-21-5p in leukemic cell-derived EVs can be introduced into T cells to induce exhaustion phenotype [24]. miR-21-5p expression in M2 macrophage-derived EVs was detected using RT-qPCR, and the results revealed that miR-21-5p expression in EVs was notably higher than that in GW4869-treated control (*p* < 0.01; Fig. 2B). Therefore, we speculated that M2 macrophage-derived EVs promoted the exhaustion of infiltrating CD8^+^ T cells in HCC through miR-21-5p. Furthermore, RNase treatment had no significant effect on miR-21-5p expression in EVs, while miR-21-5p expression was notably reduced in EVs after the combined treatment of RNase and EVs lysate (all *p* < 0.01; Fig. 2B), indicating that miR-21-5p was encapsulated in EVs. miR-21-5p expression in M2 macrophages was significantly reduced after transfection with miR-21-5p inhibitor (*p* < 0.01; Fig. 2C). Then, EVs were isolated from miR-21-5p inhibitor-transfected M2 macrophages, and miR-21-5p expression in EVs-inhi was also notably decreased (*p* < 0.01; Fig. 2D). HCC mice were treated with EVs-inhi, and miR-21-5p expression in liver tissues of mice was decreased (all *p* < 0.05; Fig. 2E), and EVs-inhi treatment partially reversed the promoting effect of EVs on CD8^+^ T cell exhaustion in primary HCC mice (Fig. 2F–I). Briefly, EVs facilitated CD8^+^ T cell exhaustion in primary HCC mice by carrying miR-21-5p.

### EVs facilitated CD8^+^ T cell exhaustion

**Fig. 3:**
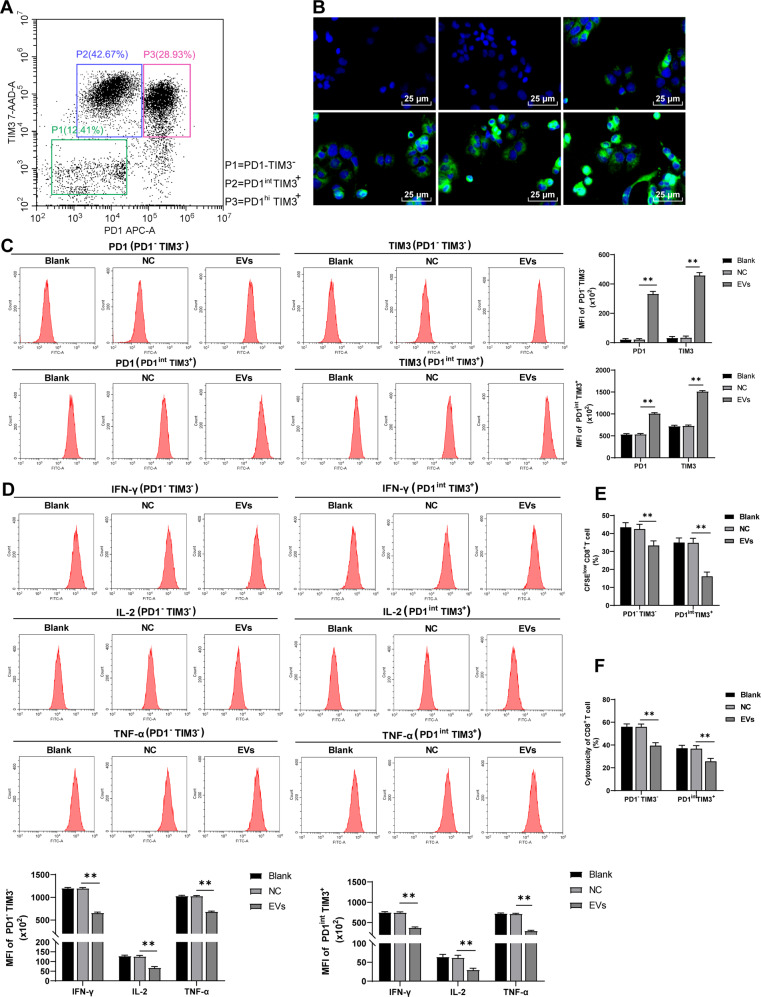
EVs facilitated CD8^+^ T cell exhaustion. **A** The infiltrating CD8^+^ T cells sorted from HCC mice were divided into three groups: normal CD8^+^ T cells (PD1^-^TIM3^-^), partially exhausted CD8^+^ T cells (PD1^int^ TIM3^+^), and completely exhausted CD8^+^ T cells (PD1^hi^ TIM3^+^). PKH67-labeled EVs were co-cultured with CD8^+^ T cells. **B** The absorption of EVs by CD8^+^ T cells was detected using immunofluorescence. **C** The expressions of inhibitory receptors in CD8^+^ T cell surface (PD1 and TIM3) were detected using flow cytometry. **D** The expressions of effector cytokines (IFN-γ, IL-2, and TNF-α) were detected using flow cytometry. **E** The proliferation ability of CD8^+^ cells was measured using flow cytometry. **F** The ability of CD8^+^ cells to kill Hep1-6 cells was measured using flow cytometry. The cell experiment was repeated 3 times. Data are presented as mean ± standard deviation and analyzed using one-way ANOVA, followed by Tukey’s multiple comparison test, **p < 0.01.

To further determine the effect of EVs on CD8^+^ cell exhaustion, the infiltrating CD8^+^ T cells sorted from HCC mice were divided into three groups: normal CD8^+^ T cells (PD1^-^TIM3^-^), partially exhausted CD8^+^ T cells (PD1^int^ TIM3^+^), and completely exhausted CD8^+^ T cells (PD1^hi^ TIM3^+^) according to the expression of PD1 and TIM3 (Fig. 3A). PD1^-^TIM3^-^ and PD1^int^ TIM3^+^ cells were selected for in vitro study. Firstly, M2 macrophage-derived EVs were labeled with PKH67 and co-cultured with CD8^+^ T cells. Under confocal microscopy, EVs were taken up by CD8^+^ T cells within 3 h (Fig. 3B). Then the effect of EVs on the function of PD1^-^TIM3^-^ and PD1^int^ TIM3^+^ cells were assessed. Consistent with the results in vivo, the EV treatment notably increased the surface expression of cell exhaustion markers on PD1^-^TIM3^-^ and PD1^int^ TIM3^+^ cells, decreased the production of effector cytokines, and weakened proliferation and the ability to kill Hep1-6 cells (all *p* < 0.01; Fig. 3C–F). In brief, EVs facilitated CD8^+^ T cell exhaustion.

### Inhibition of miR-21-5p in EVs partially reversed the promoting effect of EVs on CD8^+^ T cell exhaustion

**Fig. 4:**
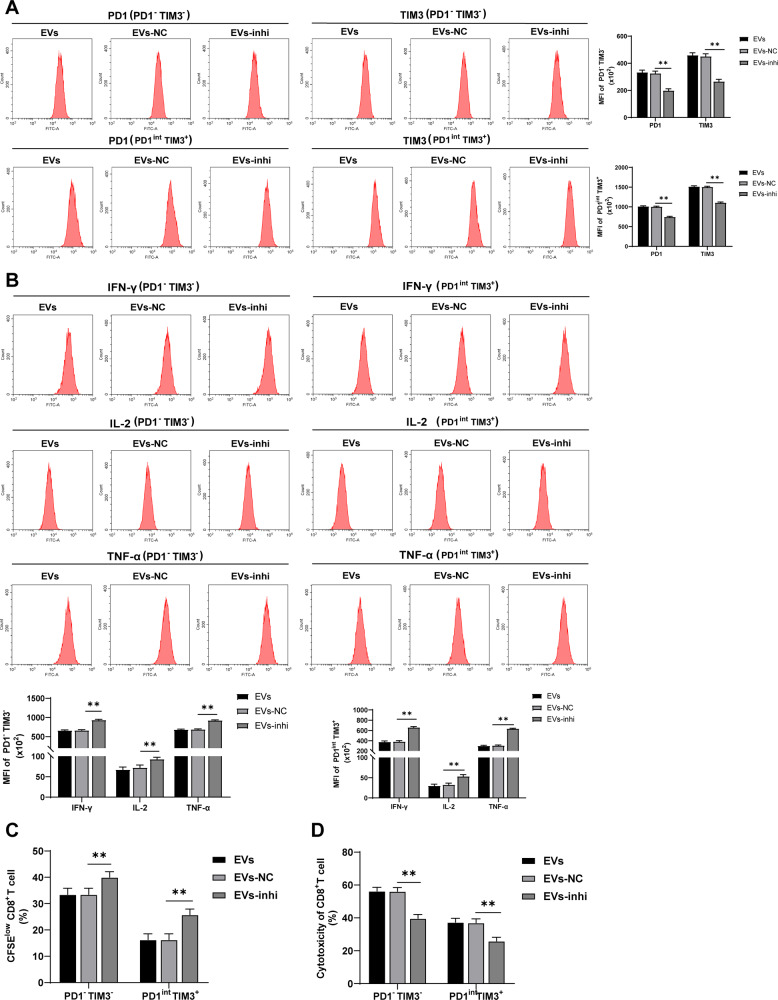
Inhibition of miR-21-5p in EVs partially reversed the promoting effect of EVs on CD8^+^ T cell exhaustion. M2 macrophages were transfected with miR-21-5p inhibitor and then EVs were isolated. The isolated EVs were co-cultured with PD1^-^TIM3^-^ and PD1^int^ TIM3^+^ cells. **A** The expressions of PD1 and TIM3 were detected using flow cytometry. **B** The expressions of IFN-γ, IL-2, and TNF-α were detected using flow cytometry. **C** The proliferation ability of CD8^+^ cells was measured using flow cytometry. **D** The ability of CD8^+^ cells to kill Hep1-6 cells was measured using flow cytometry. The cell experiment was repeated 3 times. Data are presented as mean ± standard deviation and analyzed using one-way ANOVA, followed by Tukey’s multiple comparison test, **p < 0.01.

To further determine whether M2 macrophage-derived EVs promote CD8^+^ T cell exhaustion through miR-21-5p, EVs under different treatments were co-cultured with PD1^-^TIM3^-^ and PD1^int^ TIM3^+^ cells, and then the exhaustion of PD1^-^TIM3^-^ and PD1^int^ TIM3^+^ cells was measured. The results elicited that inhibition of miR-21-5p in EVs partially reversed the promoting effect of EVs on PD1^-^TIM3^-^ and PD1^int^ TIM3^+^ cell exhaustion (all *p* < 0.01; Fig. 4A–D).

### miR-21-5p targeted YOD1

**Fig. 5:**
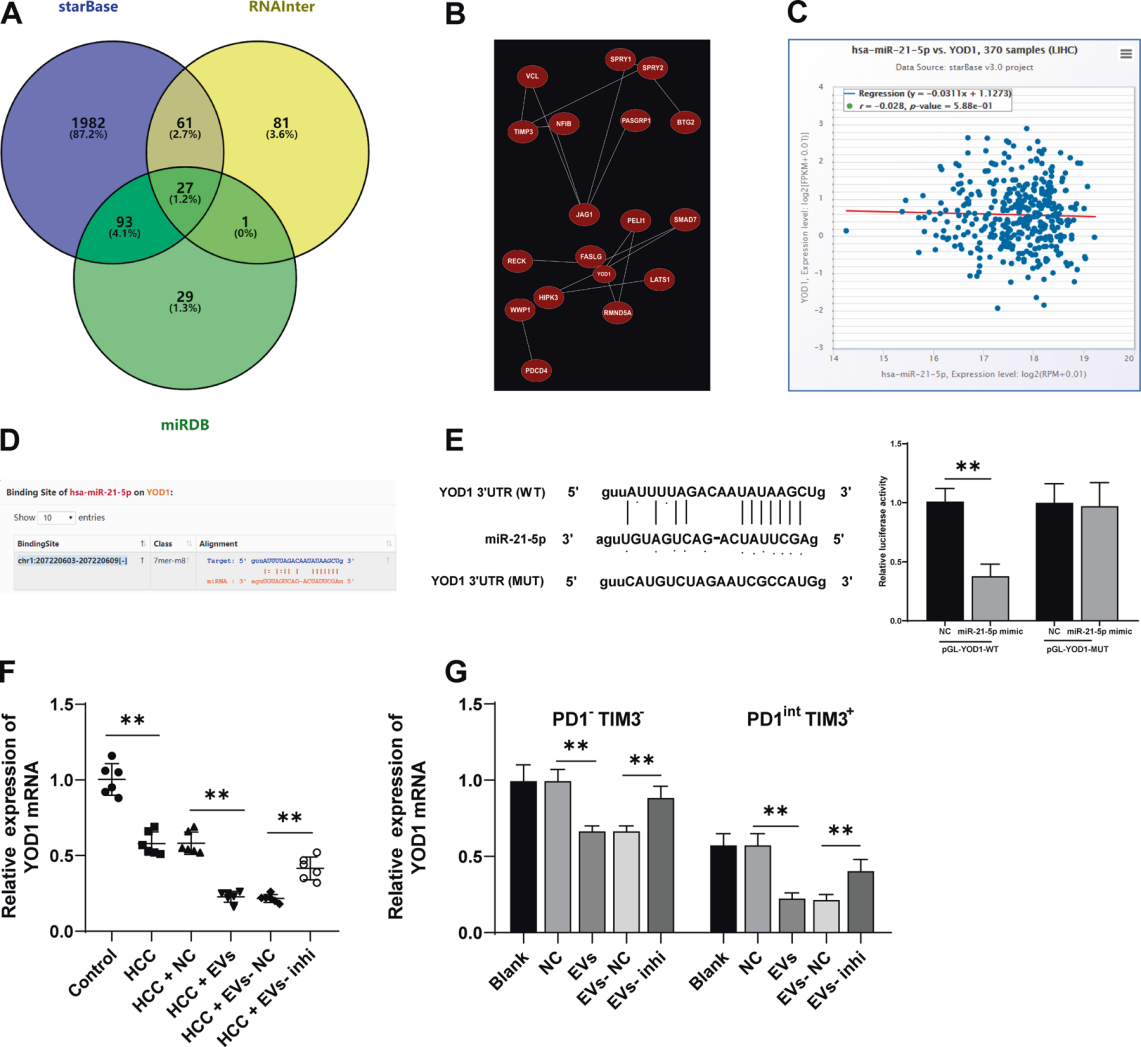
miR-21-5p targeted YOD1. **A** Venn diagram of downstream genes of miR-21-5p predicted through Starbase, RNAInter, and miRDB databases. **B** Coexpression network of candidate genes was analyzed through the Coexpedia database. **C** The correlation between the expressions of miR-21-5p and YOD1 was analyzed through the ECORI Pan-Cer database. **D** The targeting relationship between miR-21-5p and YOD1 was predicted through the StarBase database. **E** The targeting relationship between miR-21-5p and YOD1 was verified using a dual-luciferase assay. **F**, **G** YOD1 mRNA expression in mouse liver (**F**) and CD8^+^ T cells (**G**) was detected using RT-qPCR. *N* = 6. The cell experiment was repeated 3 times. Data are presented as mean ± standard deviation and analyzed using one-way ANOVA, followed by Tukey’s multiple comparison test, **p < 0.01.

To further explore the regulatory mechanism of miR-21-5p promoting CD8^+^ T cell exhaustion in EVs, the downstream targets of miR-21-5p were predicted through Starbase, RNAInter, and miRDB databases (Fig. 5A). The Coexpedia database was used to explore the coexpression network of genes to further screen target genes. According to the coexpression relationship score of the website, SPRY2 and YOD1 showed higher scores (score = 9.825, 9.200) (Fig. 5B). The antigen cross-presentation is regulated by the activity of deubiquitinase YOD1, which may help to control the immune response of antigen-specific CD8^+^ T cells in the immune process [25]. Additionally, the ECORI Pan-Cer database showed that miR-21-5p and YOD1 were negatively correlated in HCC (Fig. 5C), and there was a binding site between miR-21-5p and YOD1 3′UTR sequence (Fig. 5D). The binding relationship between miR-21-5p and YOD1 was further verified using dual-luciferase assay (*p* < 0.01; Fig. 5E). Furthermore, YOD1 expression in the liver and CD8^+^ T cells was detected. The results revealed that YOD1 mRNA expression in mouse liver and PD1^-^TIM3^-^ and PD1^int^ TIM3^+^ cells was notably decreased after EV treatment, and YOD1 mRNA expression was increased after inhibition of miR-21-5p in EVs (all *p* < 0.01; Fig. 5F, G). Taken together, there was a negative regulatory relationship between miR-21-5p and YOD1.

### Overexpression of YOD1 averted the effect of EVs on CD8^+^ T cell exhaustion

**Fig. 6:**
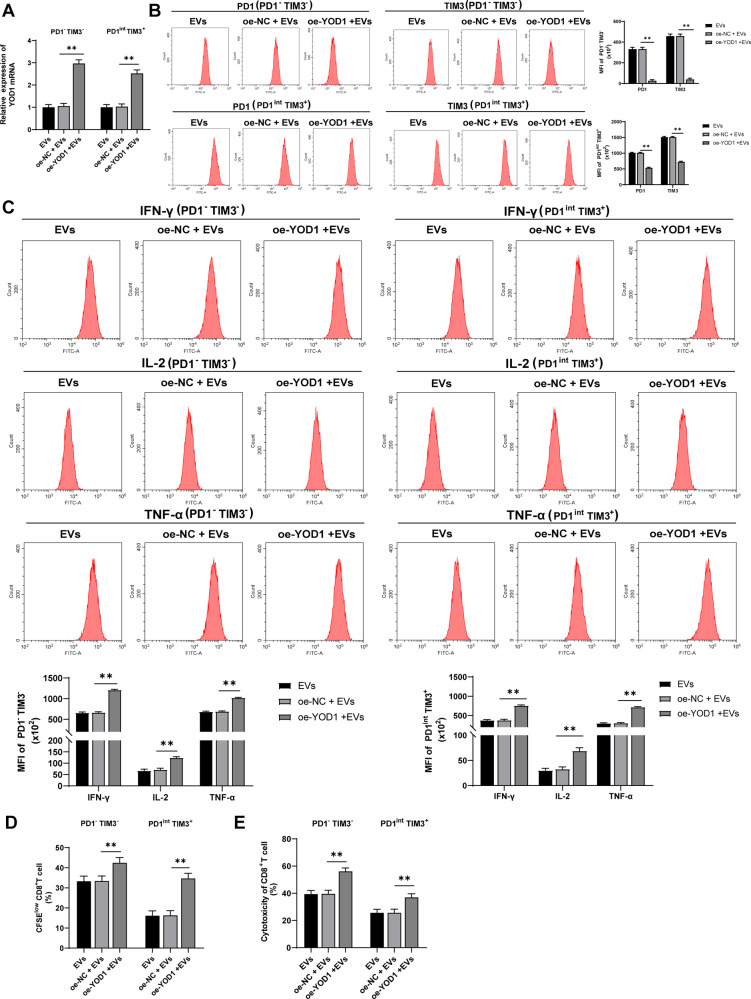
Overexpression of YOD1 reversed the effect of EVs on CD8^+^ T cell exhaustion. pcDNA-YOD1-transfected CD8^+^ T cells were treated with EVs. **A** YOD1 mRNA expression was detected using RT-qPCR. **B** The expressions of PD1 and TIM3 were detected using flow cytometry. **C** The expressions of IFN-γ, IL-2, and TNF-α were detected using flow cytometry. **D** The proliferation ability of PD1^-^TIM3^-^ and PD1^int^ TIM3^+^ cells was measured using flow cytometry. **E** The ability of PD1^-^TIM3^-^ and PD1^int^ TIM3^+^ cells to kill Hep1-6 cells was measured using flow cytometry. The cell experiment was repeated 3 times. Data are presented as mean ± standard deviation and analyzed using one-way ANOVA, followed by Tukey’s multiple comparison test, **p < 0.01.

To explore the role of YOD1 in EVs promoting CD8^+^ T cell exhaustion, PD1^-^TIM3^-^ and PD1^int^ TIM3^+^ cells were transfected with pcDNA-YOD1 and then treated with EVs. RT-qPCR showed that YOD1 mRNA expression in PD1^-^TIM3^-^ and PD1^int^ TIM3^+^ cells were increased after oe-YOD1 transfection (all *p* < 0.05; Fig. 6A). Overexpression of YOD1 reversed the promoting effect of EVs on CD8^+^ T cell exhaustion (all *p* < 0.05; Fig. 6B–E).

### YOD1 inhibited the YAP/β-catenin pathway

**Fig. 7:**
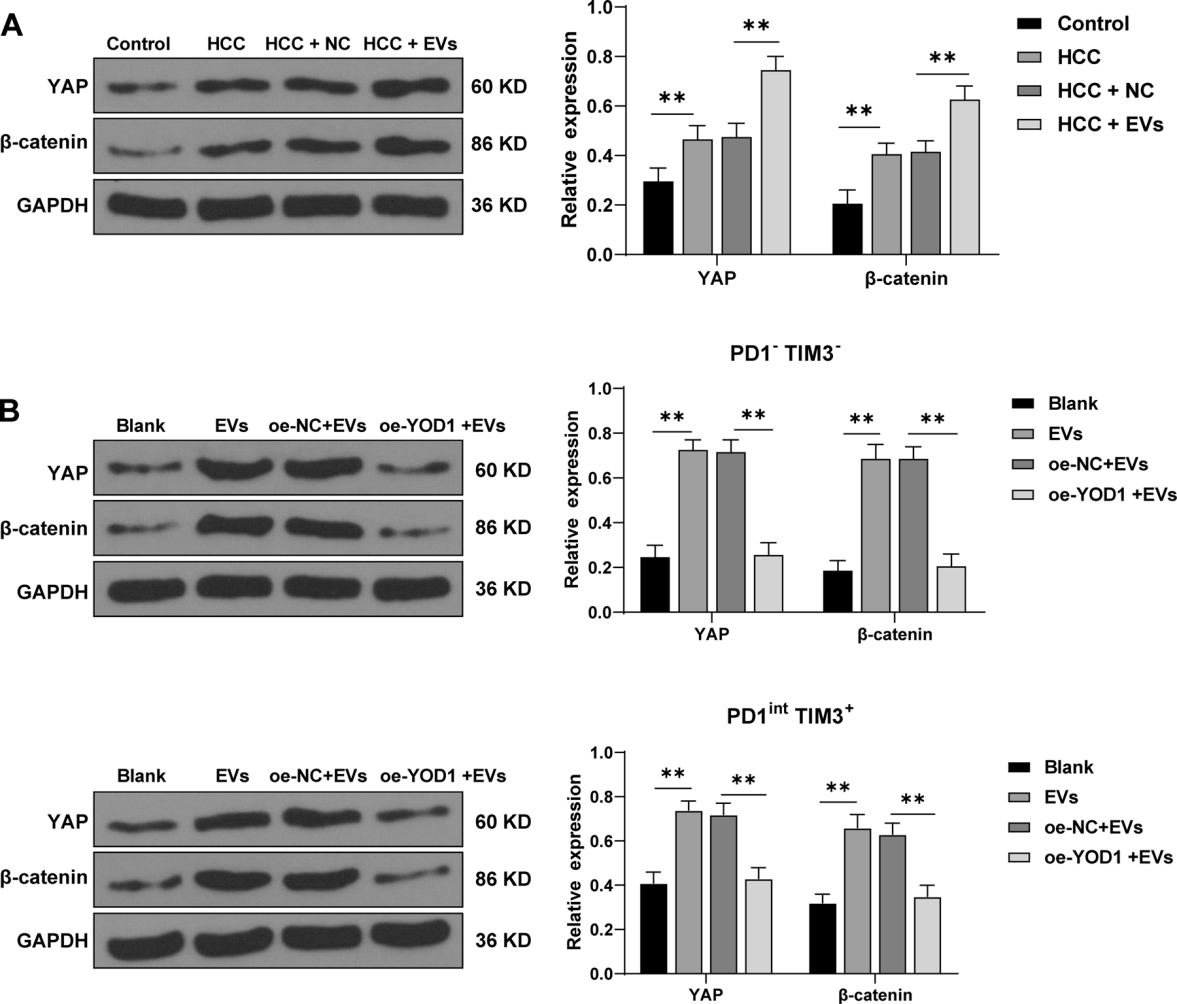
YOD1 inhibited the YAP/β-catenin pathway. **A**, **B** The levels of YAP and β-catenin in HCC mouse liver and PD1^-^TIM3^-^ and PD1^int^ TIM3^+^ cells were detected using Western blotting. *N* = 6. The cell experiment was repeated 3 times. Data are presented as mean ± standard deviation and analyzed using one-way ANOVA, followed by Tukey’s multiple comparison test, **p < 0.01.

YOD1 and YAP expressions are closely related to HCC, and YOD1 is a regulator of the Hippo pathway and a potential target for the treatment of HCC [26]. YAP in the Hippo pathway attenuates CD8^+^ T cell-mediated tumor responses [27]. Activation of β-catenin promotes immune escape in HCC cells [28]. Western blotting showed that compared with control mice, HCC mice had elevated expressions of YAP and β-catenin, and EV treatment further enhanced their expressions (all *p* < 0.01; Fig. 7A). To further investigate the effect of YOD1 on the YAP/β-catenin pathway, we overexpressed YOD1 in EV-treated PD1^-^TIM3^-^ and PD1^int^ TIM3^+^ cells. The results revealed that the expressions of YAP and β-catenin were clearly decreased after YOD1 overexpression (both *p* < 0.01; Fig. 7B). Briefly, YOD1 inhibited the YAP/β-catenin pathway.

## Discussion

**Fig. 8:**
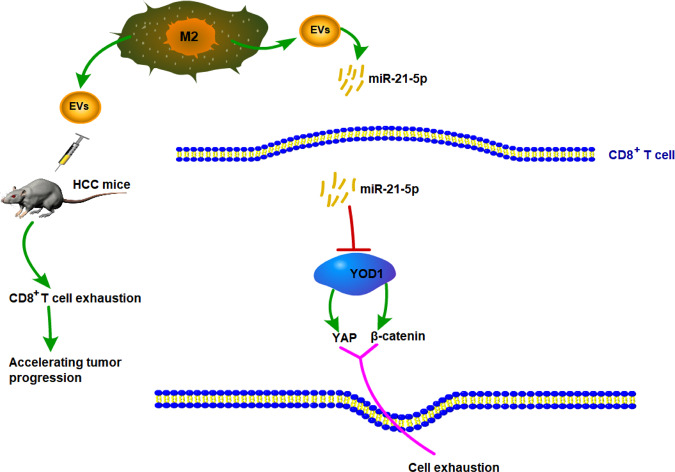
M2 macrophage-derived EVs promote the depletion of CD8 +T cells in HCC via the miR-21-5p/YOD1/YAP/β-catenin axis. M2 macrophage-derived EVs carried miR-21-5p into CD8^+^ T cells, inhibited YOD1 expression, and activated the YAP/β-catenin pathway, thereby promoting CD8^+^ T cell exhaustion in HCC.

HCC is an aggressive malignant tumor associated with unfavorable outcomes, and immune checkpoint-based therapy has been recognized to be a promising strategy for HCC management [8]. The crosstalk between HCC cells and TME components drives the immune evasion of HCC, and tumor-associated M2 macrophages are the primary components of TME [29], which facilitate the progression and invasiveness of HCC [30]. This study elucidated that M2 macrophage-derived EVs promoted CD8^+^ cell exhaustion in HCC via the miR-21-5p/YOD1/YAP/β-catenin pathway (Fig. 8).

Immune cells in TME interact with tumor cells directly or via chemokine and cytokine signalings, thus affecting tumor cell behaviors and therapeutic response [16]. M2 macrophages can enhance tumor angiogenesis, matrix remodeling, and adaptive immunity suppression [17], which are concerned with poorer outcomes and worse pathological characteristics of HCC patients [31]. After the murine model of primary HCC was established by DEN/CCl_4_ combined induction in the present study, EVs were injected into liver tissues of model mice. The results demonstrated that EV treatment promoted the tumorigenesis of mice, with a higher malignant degree of the tumor. During chronic infection and cancer progression, T cells are exposed to persistent antigen and/or inflammatory signals, leading to an exhausted status [11]. The exhausted CD8^+^ T cell constitutes a major obstacle to effective immunotherapy in HCC [10]. In this study, the number of infiltrating CD8^+^ T cells in HCC mice was significantly reduced, and EV treatment further reduced the number of infiltrating CD8^+^ T cells in HCC tissues.

The exhausted CD8^+^ T cells are characterized by the gradual loss of effector functions including cytokine production, killing function, the generation of inhibitory receptors, metabolic disorder, and homeostatic proliferation [32]. Then, we isolated PBMCs from the mouse liver and sorted CD8^+^ T cells. PD1 and TIM3 are commonly overexpressed in exhausted CD8^+^ T cells, and the intensity of inhibitory receptors is positively correlated with the degree of CD8^+^ T cell exhaustion [11]. In the current study, HCC mice showed notably enhanced expressions of immune checkpoint inhibitory receptors PD1 and TIM3 and reduced expressions of effector cytokines IFN-γ, IL-2, and TNF-α, indicating that CD8^+^ T cells in HCC mice were in a functional exhaustion state. The addition of EVs further increased the expressions of CD8^+^ cell exhaustion surface markers, decreased the production of effector cytokines, and weakened the proliferation ability of CD8^+^ cells and the ability to kill Hep1-6 cells. EVs derived from cord blood stem cells are more likely to interact with monocytes in PBMCs and induce the differentiation of purified monocytes into M2 macrophages, which can downregulate the percentage of activated CD8^+^ T cells [33]. Briefly, M2 macrophage-derived EVs promoted CD8^+^ cell exhaustion in mice with primary HCC.

It is well established that EVs can facilitate tumor progression and metastasis via the delivery of multiple molecules, including miRNAs [34]. Aberrant miRNA expression has been commonly accepted as a promising biomarker for the diagnosis and treatment of HCC [35]. miR-21 represents one of the earliest discovered oncogenes, which can target a variety of tumor suppressor genes related to proliferation, apoptosis, and invasion [36]. Emerging evidence has revealed that the elevated miR-21 expression facilitates HCC progression [37, 38]. Hence, miR-21-5p expression in M2 macrophage-derived EVs was detected, and the results revealed that miR-21-5p expression in EVs was notably increased. We speculated that M2 macrophage-derived EVs promoted CD8^+^ T cell exhaustion in HCC through miR-21-5p. M2 macrophages were transfected with miR-21-5p inhibitor and then EVs were extracted (EVs-inhi). EVs-inhi treatment partially reversed the promoting effect of EVs on CD8^+^ T cell exhaustion in mice with primary HCC. Notably, leukemia cell-derived EVs have been demonstrated to trigger T cell exhaustion via miR-21-5p delivery [24]. These results suggested that EVs facilitated CD8^+^ T cell exhaustion in mice with primary HCC by carrying miR-21-5p. Knockdown of miR-21 can enhance CD8^+^ T cell proliferation and cell cytotoxic activity in glioma [39]. miR-21 antagomir upregulated the proportion of CD4^+^ and CD8^+^ T cells in lung cancer tissues, implying that inhibition of miR-21 retards the immunosuppressive ability of myeloid-derived suppressor cells on lung cancer [40]. For the in vitro study, PD1^-^TIM3^-^ and PD1^int^ TIM3^+^ cells were cultured with EVs to further determine the effect of EVs on CD8^+^ T cell exhaustion. The results were consistent with those of the in vivo experiments and confirmed that EVs facilitated CD8^+^ T cell exhaustion by carrying miR-21-5p.

Subsequently, we screened the target genes of miR-21-5p through the databases. YOD1 is a member of deubiquitinating enzymes of Otubain domain [41]. Although it is suggested that YOD1 can act as a potential therapeutic target for liver cancer [42], the exact role of YOD1 in HCC remains controversial yet. This study verified the negative regulatory relationship between miR-21-5p and YOD1. Overexpression of YOD1 reversed the effect of EVs on CD8^+^ cell exhaustion. YOD1 provides exogenous antigens to CD8^+^ cells and thus controls antigen-specific CD8^+^ cell response during immunization [25]. Then we determined the downstream pathway regulated by the miR-21-5p/YOD1 axis in HCC. A close correlation between YOD1 and YAP expression can be observed in patients with liver cancer [42]. YAP serves as a sensor of the structural and mechanical characteristics in the cell microenvironment [43]. The strict control of YAP activity is essential for the homeostasis of normal tissues and activated YAP results in excessive tissue growth, stem cell expansion, and tumorigenesis [44]. YAP is deregulated in HCC and emerged as a promising target for HCC therapies [44]. Aberrant activation of the β-catenin pathway is frequently observed in HCC patients, which is implicated in drug resistance, tumor initiation, progression, and metastasis [45]. In this study, HCC mice showed elevated expressions of YAP and β-catenin, and EV treatment further enhanced their expressions. Furthermore, we overexpressed YOD1 in PD1^-^TIM3^-^ and PD1^int^ TIM3^+^ cells treated with EVs. The results revealed that the expressions of YAP and β-catenin were decreased after YOD1 overexpression. YAP plays an immunosuppressive role in CD8^+^ cells, especially in activated cytotoxic cells in the TME [27]. Additionally, β-catenin signaling regulates T cell inflammation and restores T cell infiltration in the TME [46]. Inhibition of YAP can restore hepatocyte differentiation in advanced HCC, thereby contributing to tumor regression [47]. Collectively, YOD1 inhibited the YAP/β-catenin pathway.

To conclude, miR-21-5p carried by M2 macrophage-derived EVs could promote CD8^+^ T cell exhaustion in HCC via targeting YOD1 and activating the YAP/β-catenin pathway. However, this study failed to investigate the role of EVs in HCC at the clinical level. Additionally, the existing study has unveiled that YAP not only functions as a tumor promoter, but also enhances the immunosuppression of CD4 and CD8 T cells [27]. YAP can typically bind to the TEAD transcription factor, but the binding protein of YAP in immune cells is still unclear. In the future, we will explore the upstream lncRNAs involved in the regulation of M2 macrophage-derived EVs or the mechanisms of other miRNAs in CD8^+^ cell exhaustion in HCC.

## Supplementary information

Supplementary Table 1

Supplementary Figure 1

## Data Availability

All the data generated or analyzed during this study are included in this published article.
