# Supplementary material for: M2 macrophage-derived extracellular vesicles facilitate CD8+T cell exhaustion in hepatocellular carcinoma via the miR-21-5p/YOD1/YAP/β-catenin pathway
Source: Cell Death Discov. 2021 Jul 16;7:182. doi: 10.1038/s41420-021-00556-3 (PMC8289864; doi:10.1038/s41420-021-00556-3)
Supplement: Supplementary file 1 — Supplementary Table 1 [file 41420_2021_556_MOESM1_ESM.docx]

**Table 1** Primer sequence for RT-qPCR

| Gene | Forward 5’-3’ | Reverse 5’-3’ |
| --- | --- | --- |
| miR-21-5p | GCAACACCAGCGAGGGC | AGTGCAGGGTCCGAGGTATT |
| YOD1 | CTTTTGCAGGGTCTTTCCAG | CAGGTCCCCAAGAGTGATGT |
| GAPDH | CTCAGACACCATGGGGAAGGTGA | ATGATCTTGAGGCTGTTGTCATA |
| U6 | ATTGGAACGATACAGAGAAGATT | GGAACGCTTCACGAATTTC |
