# Supplementary figures and images for: M2 macrophage-derived extracellular vesicles facilitate CD8+T cell exhaustion in hepatocellular carcinoma via the miR-21-5p/YOD1/YAP/β-catenin pathway
Source: Cell Death Discov. 2021 Jul 16;7:182. doi: 10.1038/s41420-021-00556-3 (PMC8289864; doi:10.1038/s41420-021-00556-3)

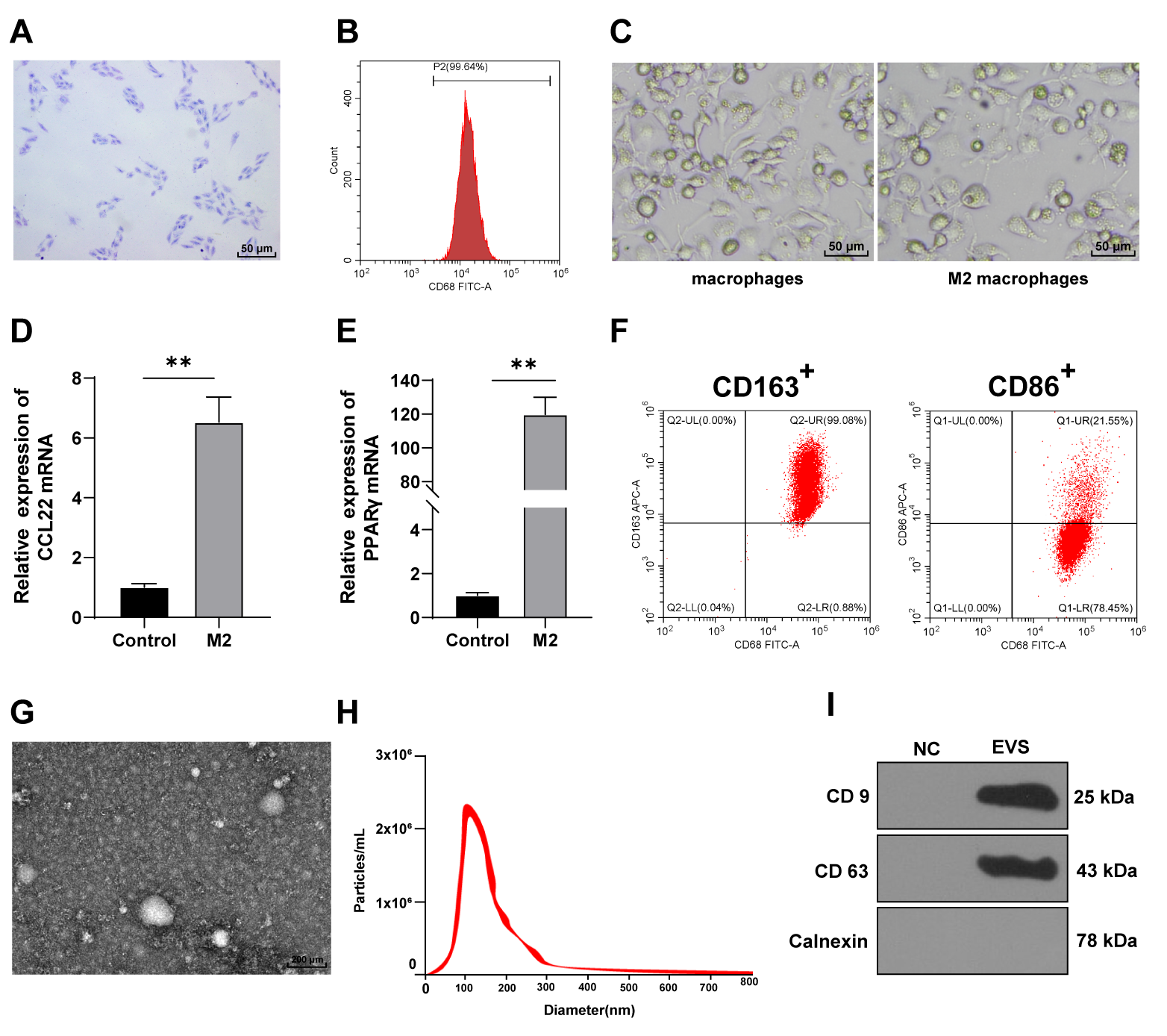

Supplement: Supplementary file 2 — Supplementary Figure 1 [file 41420_2021_556_MOESM2_ESM.tif]
